# A Neglected Issue on Sexual Well-Being following Breast Cancer Diagnosis and Treatment among Chinese Women

**DOI:** 10.1371/journal.pone.0074473

**Published:** 2013-09-25

**Authors:** Fengliang Wang, Fei Chen, Xiqian Huo, Ruobing Xu, Liang Wu, Jianming Wang, Cheng Lu

**Affiliations:** 1 Department of Breast, Nanjing Maternity and Child Health Hospital of Nanjing Medical University, Nanjing, China; 2 Department of Epidemiology and Biostatistics, School of Public Health, Nanjing Medical University, Nanjing, China; Nanjing Medical University, China

## Abstract

**Background:**

Changes to sexual well-being can be one of the most problematic quality of life issues following the diagnosis and treatment of breast cancer. The objectives of the present study were to evaluate changes to sexual well-being following breast cancer, to expand upon the existing body of knowledge pertaining to breast cancer and sexuality, and to provide the necessary information for implementing future interventions that may help improve the quality of life in breast cancer patients.

**Methods:**

This study was mixed with qualitative and quantitative designs. Twenty patients with breast cancer were recruited for in-depth interviews. The central questions covered a patient’s cancer experience and perceptions of sexual activities following breast cancer. According to the findings of the qualitative study, we performed a quantitative study using a structured questionnaire to collect data on patient’s experience and attitude to sexual well-being following breast cancer diagnosis and treatment.

**Results:**

Based on the qualitative analysis, seven main themes emerged: (1) Decrease in sexual frequency; (2) Lack of sexual interest; (3) Menopausal symptoms; (4) Body image changes; (5) Effects on marital relationship; (6) Misconceptions about sex; (7) The need for professional consultation. Results from the quantitative study further supported the findings from the qualitative analysis, where changes to sexual well-being were common following cancer diagnosis and treatment and it was a neglected issue among Chinese women.

**Conclusions:**

The present study highlights the significant changes to sexual well-being following breast cancer, in addition to the lack of knowledge and misconceptions of sexual activity among patients. Addressing these problems will help improve a patient’s quality of life. The findings of this study could help healthcare professionals recognize the sexual issues faced by women with breast cancer and ultimately promote a healthy life.

## Introduction

Breast cancer is one of the most prevalent cancers and the second-leading cause of cancer-related deaths in women worldwide [1]. Because advances in breast cancer screening and treatment have led to a significant improvement in patient survival [2], to evaluate the medical and psychosocial needs of cancer survivors is becoming increasingly important [3]. Changes in sexual well-being and sexual dysfunction are common following breast cancer diagnosis and treatment [4]–[7]. This has led breast cancer researchers to focus on life quality issues, with a particular focus on sexual well-being [8].

Following a diagnosis of breast cancer, patients are primarily concerned with personal survival and lifestyle changes that may follow the cancer. After successful treatment of breast cancer, patients tend to focus on life quality issues, such as sexual well-being [9]. Changes to sexual well-being can be one of the most problematic issues that a patient can face. It can impact a patient’s life for many years, and can be associated with serious physical and psychological effects [10]. It is estimated that 15%–64% of women with breast cancer experience symptoms of sexual dysfunction, including reduced arousal, lack of sexual desire, vaginal dryness, and dyspareunia [7], [10], [11]. Although patients often seek help for cancer related services, many may be reluctant to seek help for sexual problems [10].

Previous studies on sexuality and breast cancer have primarily used quantitative survey methods during data collection. These methods may not fully address the complexity of a woman’s living experience, cultural background, and perception of sexual well-being [8]. Therefore, we utilized a mixed study designed by combining qualitative and quantitative researches. The objectives of the present study were to evaluate changes to sexual well-being following breast cancer, expand the existing body of knowledge pertaining to breast cancer and sexuality, and provide the necessary information for implementing future interventions that may help improve the quality of life in breast cancer patients.

## Methods

### Ethical Consideration

The Institutional Review Board (IRB) of Nanjing Medical University approved the study. Written informed consents were obtained from all participants.

### Design and Data Collection

This study was designed to use both qualitative and quantitative methods in order to gain insights into patient’s sexual well-being following breast cancer diagnosis and treatment.

#### 1. Qualitative study

An exploratory, interview-based qualitative study was conducted to assess breast cancer patients’ perspectives of sexual well-being following breast cancer. Patients were identified through electronic records obtained from Nanjing Maternity and Child Health Hospital. Participants were selected based on a convenience sampling strategy according to age, marital status, number of years since cancer diagnosis, and treatment history. Researchers contacted patients through telephone, explained details of the study to each patient, and asked for each patient’s informed consent to participate in the study. If consent was given, patients were invited to the hospital to participate in a qualitative interview. Twenty patients participated in the study. All patients were residents of Nanjing City of China. Demographics of patients are listed in Table 1.

**Table 1:** Basic characteristics of participants in in-depth interviews (N = 20).

Participant	Age (years)	Maritalstatus	Career	Children	Cancer type	Number of yearssince diagnosis	Tumor size	Lymph node metastasis	Chemotherapy*	Hormonal therapy
Qian**	42	Married	Self-employed	1	Infiltrating ductal carcinoma	2	2*2.5	2/24	TH	Tamoxifen
Sun**	38	Married	Worker	1	Carcinoma in situ	3	3*3	0/16	X	No
Deng**	43	Married	Nurse	1	Infiltrating ductal carcinoma	3	2.5*1.5	0/15	EC	Tamoxifen
Liu**	48	Married	Bank staff	1	Infiltrating ductal carcinoma	2	2*1	0/19	TE	Tamoxifen
Huang**	44	Married	Worker	1	Infiltrating ductal carcinoma	0.5	2*1.5	0/19	EC-TX	No
Yin**	36	Married	Staff	1	Infiltrating ductal carcinoma	2	2*2	0/19	TH	Tamoxifen
Wang**	37	Married	Staff	1	Carcinoma in situ	2	5*5	0/19	TC	Tamoxifen
Xu**	48	Married	Worker	1	Infiltrating ductal carcinoma	10	NA	0/20	TE	Tamoxifen
Chen**	47	Married	Accountant	1	Infiltrating ductal carcinoma	2	4.5*3.5	2/30	TEC	Tamoxifen
Dai**	38	Married	Farmer	2	Infiltrating ductal carcinoma	1	0.8*0.6	0/29	FEC	No
Han**	38	Married	Unemployed	1	Infiltrating ductal carcinoma	1	3.5*2	0/12	TE	Tamoxifen
He**	46	Married	Unemployed	1	Infiltrating ductal carcinoma	2	3*2	7/17	TEC-FEC	Tamoxifen
Wang**	40	Married	Shop assistant	1	Infiltrating ductal carcinoma	1	3*2	1/19	TE	Tamoxifen
Wang**	37	Married	Unemployed	1	Infiltrating ductal carcinoma	1	2.5*2	0/22	TE	Tamoxifen
Liu**	49	Married	Worker	1	Infiltrating ductal carcinoma	1	1.3*1	0/13	CEX	Tamoxifen
Zong**	48	Married	Store keeper	1	Infiltrating ductal carcinoma	0.5	1.5*1	0/14	EC-T	Tamoxifen
Yang**	48	Married	Farmer	2	Infiltrating ductal carcinoma	2	2.5*2.5	0/5	FEC	No
Wan**	48	Married	Logistic staff	1	Infiltrating ductal carcinoma	3	3*3	0/29	TE	Tamoxifen
Lv**	47	Divorced	Unemployed	1	Infiltrating ductal carcinoma	3	4*3	3/19	TE	Tamoxifen
Ye**	50	Married	Accountant	1	Infiltrating ductal carcinoma	4	2.5*2.5	2/17	NE	Tamoxifen & Goserelin

* T: Docetaxel; X: Capecitabine; E: Epirubicin; C: Cyclophosphamide; H: Trastuzumab; F: Fluorouracil; N: Vinorelbine.

In-depth interviews were designed and based on the concepts from “Interviews: An Introduction to Qualitative Research Interviewing” [12]. At the beginning of each interview, the researchers explained the purpose of the study to the participants. An interview guide helped the interviewer to focus on important topics, maintain consistency across interviews, and remain on topic during the interview process. The central questions of the interview covered a patient’s cancer experience and perceptions of sexuality following breast cancer. Interview questions were developed based on a literature review, and the clinical experience of the research team. Central questions included: (1) What changes to your sexuality and sexual well-being have you noticed following breast cancer diagnosis and treatment; (2) What is the attitude of you and your partner to the sex issues following breast cancer diagnosis and treatment; (3) How do changes to your sexuality and sexual well-being influence the quality of life; (4) What are the perceived causes of these changes; (5) Have you received professional counseling for sexual problems following breast cancer; (6) Where do you acquire information about sexual issues following breast cancer; (7) Do you require or wish to seek clinical consultation related to sexual issues. The above-mentioned topics were covered in all interviews. Researchers also encouraged the discussion of other topics related to sex that each patient considered significant. The in-depth interviews were conducted in Nanjing Maternity and Child Health Hospital in Mandarin Chinese language. Each interview lasted approximately 20–40 minutes. All interviews were audio taped with participant permission. Interviewers guided the respondent through conversation until all of the critical issues listed on the interview guide were inquired. The research team included clinical doctors and academic researchers. FW and FC are highly experienced in clinical and research fields of breast cancer; CL is a breast surgeon with experience in the psychological consultation of patients with breast cancer, and JW is an academic researcher specialized in epidemiology and health services. All members of the research team discussed regularly, contributing to study design, implementation, and data analysis.

#### 2. Quantitative study

Following the qualitative analysis, we performed a field investigation in Zhenjiang city. Two hundred patients with breast cancer were randomly selected from the cancer registry book with the following inclusion criteria: (1) Patients were aged between 30 to 60 years; (2) They were diagnosed and treated with breast cancer during 2007 and 2012; (3) All of them were married women and lived together with their spouses. We used a structured questionnaire to collect data on patients’ experiences and attitude towards sex following the diagnosis and treatment of breast cancer. The questions were designed based on the findings from the qualitative study. Patients were interviewed by trained clinical doctors through telephone or at the hospital.

### Analysis

#### 1. Qualitative study

Interviews were analyzed aiming to “offer insight, enhance understanding and provide a meaningful guide to action” [13], [14] . In addition to recording each interview, interviewers kept field notes documenting their immediate perceptions of the session, in addition to documenting nonverbal data. The recorded interviews were transcribed verbatim and later compared with the audio recordings to ensure the accuracy of the transcripts. The researchers continuously examined the data and highlighted important points within the text or wrote comments in the margins. Transcripts and notes were analyzed thematically by creating coding nodes for common themes and subthemes. Codes were developed by FW and JW, and were based on the original terms used by the participants. The interviewers independently coded the transcripts line by line. Codes were compared among interviewers and discussed during regular meetings within the research team (FW, FC, XH, XX, LW, JW, and CL) until a consensus code could be reached. Tentative categories and sub-categories were created from the clustered codes. Subsequently, major themes emerged from the clustered codes that were based on the patterns and relationships between the categories. The categories were considered saturated when no new themes were revealed [13], [15]. The adequacy of emerging themes and thematic relationships was interpreted by the authors (FW, CL and JW), in consultation with other team members (FC, XH, RX and LW). In addition to regular team meetings regarding data analysis and triangulation, rigor was ensured by having study participants review a written summary of our findings. Participants were asked how the researcher-developed themes compared with their own perspectives and experiences, to ensure that an accurate representation of the participants’ perspectives had been sustained. Participants could also clarify their initial statements, add information, and prioritize the initial themes [16]. Three masters-level graduate students also provided a unique triangulation to cross-examine our results. The students had no previous experience working with patients with breast cancer and therefore asked questions from the perspective of a novice to both the data and the analytic method, which allowed for analyst triangulation [17]. Additional validation was obtained through discussions with clinical researchers regarding our findings. Triangulation procedures were conducted until further analyses did not lead to additional emerging themes. In this manuscript, all quotations from participants are indicated in italics, and participants are identified within the text by pseudonym, age, marital status, and the number of years since diagnosis.

#### 2. Quantitative study

Data were entered in Epidata 3.1 (Denmark) and analyzed using STATA 10.0 (College Station, TX, USA). Patient’s experience and attitude towards sexual issues were described with proportions.

## Results

### Findings from the Qualitative Study

Twenty women with breast cancer participated in this study. The mean (±S.D.) age of the study subjects was 43.6 (±4.9) years, and the mean time since diagnosis was 2.3 years (Table 1). Eighteen patients (90%) were diagnosed with infiltrating ductal carcinoma and two patients (10%) were diagnosed with carcinoma in situ. All patients had received chemotherapy, while 80% had received hormonal therapy. Tamoxifen was the most commonly used drug for breast cancer treatment among our study participants. Based on the qualitative analysis, seven main themes emerged: (1) Decrease in sexual frequency; (2) Lack of sexual interest; (3) Menopausal symptoms; (4) Body image changes; (5) Effects on marital relationship; (6) Misconceptions about sex; (7) The need for professional consultation.

#### 1. Decrease in sexual frequency

Sexual dysfunction was highly prevalent among women with breast cancer. Eighteen out of twenty patients reported a significant reduction in the frequency of sexual activities following diagnosis of breast cancer. Nearly half patients responded that they had abstained from intercourse following breast cancer, and some patients indicated they only engaged in sexual activities one or two times per year. “Since the diagnosis of breast cancer, I have never had sex with my husband. The reasons include lack of desire, inability to relax, painful intercourse and vaginal dryness. As I know, many patients face the same problem. There is a significant decrease in sexual frequency among breast cancer patients.” (Yin **, 36 years old, married, 2 years after diagnosis).

#### 2. Lack of sexual interest

A significant proportion of patients reported depression and the loss of sexual desire following cancer diagnosis. One patient described, “Now I only care about my health and do not expect too much from sex. I have no desire for having a sexual life. The sexual interest has totally disappeared” (Liu **, 48 years old, married, 0.5 years after diagnosis). Another patient reported, “After the diagnosis of breast cancer, the frequency of sexual activities has decreased significantly. Now I am not enthusiastic about sex and sometimes I even think intercourse is a terrible thing. The only reason for having sex with my partner after the breast cancer is that I am worried about the marital relationship” (Deng **, 43 years old, married, 3 years after diagnosis). For some patients who have continued sexual relations with their partners, the quality of the sexual experience seemed to have declined considerably. “The feeling is worse, and it is not exciting now. Though I have no sexual desire, I can’t refuse to have sex with my husband as he takes very good care of me” (He **, 46 years old, married, 2 years after diagnosis).

#### 3. Menopausal symptoms

A frequent complaint of women following breast cancer treatment is the appearance of menopausal-like symptoms. These symptoms could affect sexual function, including vaginal dryness, irritability, mood swings, and loss of tissue elasticity. Patients complained that chemotherapy induced menopausal changes, such as vaginal dryness, that made sexual intercourse more challenging. “Since the initiation of chemotherapy, my vagina has turned drier and drier. I am afraid of having sex with my husband now. Fortunately, he worked in another city last year. When he returned in January this year, he tried to have sex with me. I felt extreme pain, which was similar to my first experience of intercourse when I got married. Then, I said ‘stop’. We haven’t had sex since then” (Chen **, 47 years old, married, 2 years after diagnosis).

#### 4. Body image changes

Altered body image and dissatisfaction with appearance due to mastectomy also affected sexuality and were major concerns for some women. Women can become self-conscious about their breasts following breast cancer simply because breast cancer affects an intimate part of the female body. “One of my breasts has been removed due to cancer. Now I have a feeling of losing something on one side of my chest. I feel uncomfortable if my husband touches my chest. It has considerably influenced my interest in sexual activity” (Han **, 38 years old, married, 1 year after diagnosis). In many cases, it is the treatment that causes the emotional scars, not the cancer itself. “One day, I asked my husband to take a look at my chest. He was frightened to see the scars on my chest due to the mastectomy. Now he dares not to see or touch my naked chest. I believe that it has influenced the sexual relationship between us” (Zong **, 48 years old, married, 0.5 years after diagnosis).

#### 5. Marital relationship

Changes in sexual well-being not only influenced the patients’ quality of life, but also influenced the patients’ relationships with their partners and led to marital distress. “In 2009, I was diagnosed with breast cancer. Since then, I have never had sex with my husband. Though my husband has the desire, I refuse to have sex with him as I have no sexual interest and desire. The conflict is obvious. As a result, we got divorced in 2011” (Lv ***, 47 years old, divorced after breast cancer, 3 years after diagnosis). One patient mentioned, “There is a female patient living in my neighborhood. After the diagnosis of breast cancer, she refused to have sex with her husband. Finally, they got divorced due to the sexual issues. I am worried whether it will happen in my family” (Xu **, 48 years old, married, 10 years after diagnosis).

#### 6. Misconceptions about sex

Patients lacked accurate knowledge pertaining to sexual issues following breast cancer. Patients were typically warned by the elderly not to have sex with their husbands because it might affect their health. Traditional views in China, particularly in rural China, suggest that women should avoid sexual activity following breast cancer diagnosis and treatment. “I had no sexual activity after breast cancer. Life is more important than sex. My husband wanted to have sex with me, but I refused, which led to a severe quarrel between us. One reason is that I have become weak after chemotherapy. Another reason is that I think sexual activities will increase the secretion of estrogen or androgen, thus influencing my health. So I didn’t dare to have sex with my husband until now” (Zong **, 48 years old, married, 0.5 years after diagnosis). Patients often do not know if it is safe to have sex following breast cancer and usually receive uneducated suggestions from their friends or elderly people regarding sexual activity. “Relatives and friends told me that sexual activity should be avoided. It will affect the breast and lead to the recurrence of the disease” (Wan **, 48 years old, married, 3 years after diagnosis). “Since the diagnosis of breast cancer, I have never had sex with my husband. We do not know whether we can have sex. There is another breast cancer patient living next to my apartment. She is only one year older than I am. Her mother told me that she has slept separately from her husband because sex is thought to be harmful to breast cancer patients. I do not know whether it is true or false. I think I need to follow their advice” (Huang **, 44 years old, married, 0.5 years after diagnosis).

#### 7. The need for professional consultation

Patients often felt that they were in need of professional consultation. However, few clinical doctors gave professional advice on sex-related issues. The most common recommendation given by doctors was to avoid pregnancy, rather than how to solve the sexual issues. Patients were eager to get the guide for sex after breast cancer. One patient expressed her needs for professional consultation, “*In fact, I always wanted to know whether I can have sex after breast cancer. However, I am ashamed to open my mouth. Only my mother talked with me about this issue. I was cautioned to cut down the frequency of sexual activity after breast cancer diagnosis. Since the initiation of chemotherapy, I never let my husband touch me. I am worried about the adverse effects of intercourse*” (Chen **, 47 years old, married, 2 years after diagnosis). Despite the increased demands of patients, professional consultation available from clinical doctors was insufficient. Patients typically sought helps from various non-professional sources. “When we visit the clinics, neither doctors nor nurses actively give suggestions on sex to us. Patients usually exchange information with each other or seek it on the Internet. We are ashamed to talk about sex with other people as we are afraid that they will laugh at us” (Lv ***, 47 years old, divorced after breast cancer, 3 years after diagnosis).

### Findings from the Quantitative Study

We contacted 200 patients through telephone and 180 (90%) responded and were successfully investigated. The average age was 48±10 years. As showed in Table 2, 88.9% patients experienced long-term sexual problems following breast cancer diagnosis and treatment. The main sexual issues that patients have faced included body image changes after mastectomy (83.3%), absence of desire (63.9%), and vaginal dryness or painful sex (50.0%). Among those facing such sexual problems, only 70.6% actively sought external helps or information. Patient’s knowledge about sex mainly came from other breast cancer patients (57.2%), friends (44.4%), or the Internet (30.0%). Patient’s opinions to sexual activity varied greatly, where 11.7% thought that sex should be abstained, as it was harmful to them. Nearly 92% patients reported their desires for professional consultation on sexual issues following cancer diagnosis and treatment.

**Table 2:** Patient’s experience and attitude towards sexual issues (N = 180).

Questions	N	%
1. Did you experience long-term sexual problems following breast cancer diagnosis and treatment?		
No	20	11.1
Yes	160	88.9
2. What are the main sexual problems you have experienced?		
Absence of desire	115	63.9
Vaginal dryness and painful sex	90	50.0
Body image changes after mastectomy	150	83.3
Others	80	44.4
3. Did you actively seek external helps or information for these sexual issues?		
No	53	29.4
Yes	127	70.6
4. If yes, where did you seek helps or information (multiple choices)?		
Doctors	11	6.1
Mother	32	17.8
Friends	80	44.4
Other patients	103	57.2
Internet	54	30.0
Other sources	49	27.2
5. What is your own opinion on sexual activity among breast cancer patients?		
Sex should be abstained as it is harmful to patient’s health	21	11.7
Depending on husband’s demand	77	42.8
Depending on my own demand	56	31.1
Don’t know	26	14.4
6. Do you need professional consultation on sexual issues following cancer diagnosis and treatment?		
No	15	8.3
Yes	165	91.7

## Discussion

Sexuality and sexual well-being among patients with breast cancer have been long neglected issues in clinical and research settings in many countries [18]. Research on sexuality and cancer among women in Asia is extremely scarce. Our results support that changes to sexual well-being following the diagnosis and treatment of breast cancer are common among breast cancer patients in the study sites in China. This is a neglected issue within clinical settings and the current service for professional counseling is insufficient.

One of the strengths of this study is the combined use of quantitative and qualitative methods in the same study. Mixed methods research has emerged alongside qualitative and quantitative approaches as an important tool for health service researchers [19]. Methodologically sound mixed methods research can improve our understanding on health issues by providing a more comprehensive picture than either method can alone [12]. This can enrich and improve our understanding and foster fresh ideas, in order to give answers to questions that are difficult to answer by a sole classical method (quantitative or qualitative).

Sexual dysfunction encompasses a broad spectrum of sexuality-related issues and involves both the psychological and physical realms [20]. It is not surprising that sexual dysfunction affects up to 90% of women being treated for breast cancer; some reports suggest that nearly all women have some forms of sexual dysfunction following breast cancer treatment [21]. Our study confirmed that sexual dysfunctions were highly prevalent among women with breast cancer. Sexuality is an important aspect of the quality of life. However, none of the patients we interviewed had received a formal sexual consultation following the diagnosis. While patients showed a strong desire for acquiring information on sex following breast cancer diagnosis, many people in China are ashamed to actively discuss sexuality due to traditional cultures [22]. In this study, we found that patients usually sought help or acquired information on sexual issues from other patients or the Internet. The lack of professional consultation has been an important issue following the clinical treatment for breast cancer. The lack of privacy and external support may hinder patients from discussing sexual issues during their clinic visits or during hospitalization. Because sexuality is considered a private topic and is not openly discussed in the community in China, oncologists and oncology nurses could adopt sexuality issues as a component of routine oncology care. Care and consultation between the breast cancer patient, her partner, the oncological team, and the primary care practitioner are recommended when formulating individualized treatment plans that minimize risk and maximize sexual wellness [23]. Patients may benefit from opportunities to talk with each other and with health professionals about the complicated issues of sexuality and sexual wellness [24], [25].

Various factors can cause changes in sexual well-being in breast cancer patients. Alterations in body image, changes in sexual self-esteem and self-efficacy, vulvovaginal atrophy owing to chemotherapy or adjuvant hormone therapy, and loss of libido are common in breast cancer survivors [23]. Surgery can affect body image through scarring or mastectomy, which can subsequently alter sexual function [10]. In this study, we only recruited patients treated with a mastectomy because breast-conserving therapy (BCT) is not frequently performed in China [26]–[28]. In many cultures, a woman’s breasts are considered a symbol of femininity and sexuality. Thus, breast loss following mastectomy can cause a variety of psychosocial problems. We noticed that women with breast cancer usually expressed dissatisfaction with their bodies following mastectomy. This observation is consistent with previous reports that women who had a mastectomy were more likely to report that breast cancer had a negative impact on their sex lives and suggests that the cause of this impact may be through changes in their body image [24]. Some women decide to use a prosthesis that fits inside a bra to fill out their clothes. Other women, specifically younger women, may opt for reconstructive surgery [29].

Despite their high prevalence, sexuality issues among breast cancer patients are detected and treated in only a small number of patients [30]. Treating sexual dysfunction requires multiple approaches involving education, treatment of underlying conditions and lifestyle modifications [20]. Several studies have shown that non-estrogenic vaginal lubricants seem to moderately decrease the occurrence of vaginal dryness and dyspareunia [31]. Educating patients relative to vaginal lubricants and moisturizers can reduce the morbidity of vaginal atrophy. Dedicating a small amount of time to educate female cancer survivors about methods to promote vaginal health can result in the reduction or elimination of vaginal discomfort [32]. When breast cancer treatment is planned, it is important to provide information about the possible effects of treatment on sexual activity, body image, sexual functioning, and the patient’s sexual relationships. Programs and interventions should be developed to help women solve the sexual problems that emerge after breast cancer treatment [24].

It is widely accepted that a multimodal approach is needed to help couples cope with the sexual problems and marital relationship challenges following breast cancer diagnosis and treatment [23]. Support from the partner can play a key role in a woman’s emotional adjustment to breast cancer [33]. Inclusion of partners (husbands) in the sample would be useful to examine the interactive dynamics of sexual well-being following breast cancer. Previous studies have revealed that the partners of patients with breast cancer presented higher levels of anxious and depressive symptomatology; poor social, psychological, and physical quality of life; and higher levels of intimacy [13]. It would also be useful to use standardized questionnaires to assess the impact of psychological well-being and the association of changes in sexual well-being with relationship dynamics and satisfaction. Future research in this area could address some issues not discussed in the present study.

## Conclusion

The present study demonstrated that changes to sexual well-being were common following breast cancer diagnosis and treatment. Patients had insufficient knowledge about sexuality and sexual well-being, despite showing a keen desire for professional consultation. Addressing these problems is important for improving the quality of life of women with breast cancer. An effective program focusing on sexual issues following breast cancer diagnosis and treatment is essential for both health care professionals and patients.
